# Effects of major vein blockage and aquaporin inhibition on leaf hydraulics and stomatal conductance

**DOI:** 10.1098/rspb.2019.0799

**Published:** 2019-06-05

**Authors:** Hisanori Harayama, Mitsutoshi Kitao, Evgenios Agathokleous, Atsushi Ishida

**Affiliations:** 1 Hokkaido Research Center, Forestry and Forest Products Research Institute, 7 Hitsujigaoka, Toyohira-Ku, Sapporo, Hokkaido 062-8516, Japan; 2 Institute of Ecology, Key Laboratory of Agrometeorology of Jiangsu Province, School of Applied Meteorology, Nanjing University of Information Science and Technology, Nanjing 210044, People's Republic of China; 3 Center for Ecological Research, Kyoto University, Otsu, Shiga 520-2113, Japan

**Keywords:** aquaporin, leaf hydraulic conductance, major vein density, stomatal conductance, vein architecture

## Abstract

The density and architecture of leaf veins determine the network and efficiency of water transport within laminae and resultant leaf gas exchange and vary widely among plant species. Leaf hydraulic conductance (*K*_leaf_) can be regulated by vein architecture in conjunction with the water channel protein aquaporin. However, our understanding of how leaf veins and aquaporins affect leaf hydraulics and stomatal conductance (*g*_s_) remains poor. By inducing blockage of the major veins and inhibition of aquaporin activity using HgCl_2_, we examined the effects of major veins and aquaporins on *K*_leaf_ and *g*_s_ in species with different venation types. A vine species, with thick first-order veins and low vein density, displayed a rapidly declined *g*_s_ with high leaf water potential in response to vein blockage and a greatly reduced *K*_leaf_ and *g*_s_ in response to aquaporin inhibition, suggesting that leaf aquaporins are involved in isohydric/anisohydric stomatal behaviour. Across species, the decline in *K*_leaf_ and *g*_s_ due to aquaporin inhibition increased linearly with decreasing major vein density, possibly indicating that a trade-off function between vein architecture (apoplastic pathway) and aquaporin activity (cell-to-cell pathway) affects leaf hydraulics.

## 1. Introduction

Plants cannot survive without water, and it is crucial to plants for water to be transported to the leaves; therefore, plant hydraulic properties strongly influence plant performance [1–3]. Leaves account for 30% or more of whole-plant hydraulic resistance, constituting an important hydraulic bottleneck [4,5]. Leaf hydraulic conductance (*K*_leaf_ = inverse of hydraulic resistance) has a strong influence on stomatal conductance (*g*_s_) and photosynthetic capacity (*A*_max_) [6,7], and ultimately on plant ecology [8–10]. Leaf venation, whose architecture varies widely among plant species [11], forms the transport network for water within a lamina and thus affects *K*_leaf_ [5]. Water flow in the xylem of a leaf vein has lower resistance per length than water flow outside the xylem between xylem and the intercellular space. Therefore, in comparison to leaves with low vein density, leaves with a high vein density have a shorter path length of outside xylem, potentially leading to higher *K*_leaf_ and *A*_max_ [6,12]. The ratio of hydraulic resistance inside and outside the xylem in a whole leaf varies among species [5]. Among the 10 species studied in a tropical rain forest, on average, 50% of the resistance was from inside the veins and 39% from outside the veins; in comparison to species that were not sun-adapted, those that were sun-adapted had a higher proportion inside the xylem [13].

While a steady-state maximum *K*_leaf_ strongly correlates with *g*_s_ and *A*_max_ among species, *K*_leaf_ is not constant and is highly dynamic in response to environmental stimuli [5]. During drought, for example, a decrease in leaf water potential (*Ψ*_leaf_) can result in embolism formation in the xylem of leaf veins, thereby reducing their hydraulic conductivity and *K*_leaf_ [14,15]. Leaf vascular redundancy (i.e. high vein density) can enhance tolerance against hydraulic disfunction in veins because water can flow around the dysfunctional vein through nearby functioning veins [16]. In addition, hydraulic conductance outside the xylem can also be modified [5]. One of the main pathways of the outside xylem is a cell-to-cell pathway through cell membranes, regulated by water channel proteins called aquaporins [17,18]. Aquaporin gene expression in a leaf can potentially be stimulated by light [19], defoliation [20] and rewatering after drought stress [21–23], resulting in an increase in *K*_leaf_. A decline in *K*_leaf_ associated with the deactivation of aquaporins in response to environmental stimuli can result in a decline in *g*_s_ [24]. Accordingly, *K*_leaf_, and thus *g*_s_, are regulated by the network structure of leaf veins and water channel aquaporins; however, the mutual involvement of leaf veins and aquaporins in *K*_leaf_ and *g*_s_ remains poorly understood.

**Figure 1.**
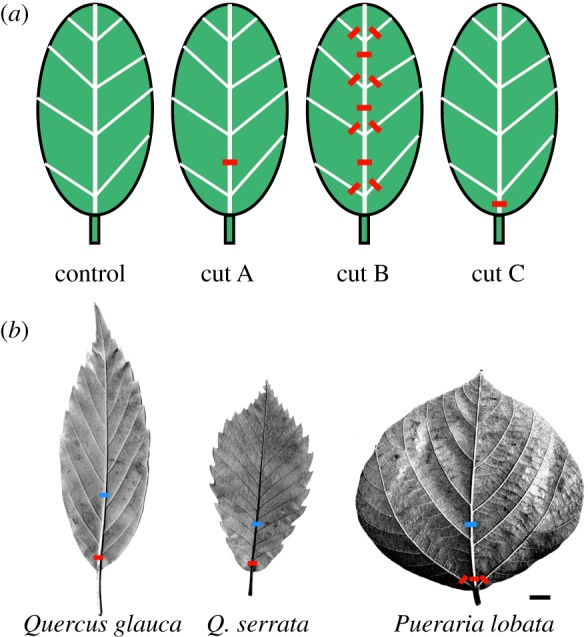
(*a*) Experimental vein blockage design according to Nardini & Salleo [25] and (*b*) images of laminae from study species. Cut surfaces indicated by red and blue lines were sealed with cyanoacrylate. (*a*) Control: intact leaves. Cut A: the midrib was cut at a quarter of the length. Cut B: the midrib was cut at three points, and all second-order veins were cut approximately 4 mm from their base. Cut C: the midrib was cut approximately 2 mm from its base; in *Pueraria lobata*, the large two second-order veins nearest to the base of the lamina were additionally cut. (*b*) Blue and red lines indicate cutting points in cuts A and C, respectively. A bar represents 1 cm.

In this study, we conducted two experiments to artificially decrease *K*_leaf_ by blocking or inhibiting water flow through major veins and aquaporins in five species with various leaf vein densities and architectures. In the major vein blocking experiments, where three vein blockage patterns were applied to mimic the embolisms in major veins with different intensities [25] (figure 1), we investigated the response of *g*_s_ with respect to *Ψ*_leaf_ in dehydrated leaves in an open field. In the aquaporin inhibition experiments [26], we investigated the decrease in *K*_leaf_ and *g*_s_ by aquaporin inhibition in fully hydrated leaves in a laboratory set-up. By integrating the results of the two experiments, we discuss the effects of leaf aquaporins and leaf venation on the interspecific difference in leaf hydraulics and *g*_s_ from physiological and ecological points of view. We hypothesized that species with lower vein density may experience greater impacts of *K*_leaf_ and *g*_s_ on aquaporin inhibition because a low vein density can provide a sensitive *K*_leaf_ response against hydraulic disfunction in veins [16], and aquaporin upregulation can offset the decline in *K*_leaf_. This hypothesis may contribute to delayed *g*_s_ reduction and prolonged photosynthesis (higher C acquisition) in vulnerable leaves with low vein density and may be connected to the trade-off function between vein architecture and aquaporin activity. The study species had different arrangements of thick leaf veins (i.e. pinnate and pinnipalmate venation; figure 1*b*), which can differentially affect *K*_leaf_ and *g*_s_ responses to hydraulic failure in the midrib of leaves [16]. The *Quercus* genus was also included because some *Quercus* species have shown an irradiance-dependent increase in *K*_leaf_ [27–29], which was related to the expression of PIP1 (Plasma membrane Intrinsic Protein) aquaporin [29].

## 2. Material and methods

Two sets of experiments, major vein blockage and aquaporin inhibition, were conducted using fully expanded current-year leaves from sunlit shoots of trees or vines grown in the arboretum of the Forestry and Forest Products Research Institute, eastern Japan (36°00′ N, 140°08′ E, 20 m.a.s.l. [30]). We studied five angiosperm species, two evergreen tree species (*Quercus acuta* Thunb. and *Quercus glauca* Thunb.) and two winter deciduous tree species (*Castanea crenata* Siebold et Zucc. and *Quercus serrata* Murray) trees and a winter deciduous vine species (*Pueraria lobata* [Willd.] Ohwi), all common in the warm-temperate forests of Japan. The leaf longevities of the evergreen *Q. acuta* and *Q. glauca* were 26 and 14 months, respectively [30]. Two to four individual trees were studied. Leaves from the vine were selected at least 5 m apart from each other because individual vines could not be identified. All trees were 20–25 years old, and their height and diameter at breast height were 10–15 m and 20–30 cm, respectively. ‘Leaf’ refers to ‘leaflet’ of the compound leaf of *P. lobata*, for brevity.

### (a) Measurement of leaf hydraulic conductance

A vacuum chamber method [31–33] was used to measure *K*_leaf_. Briefly, a leaf was placed in a vacuum chamber (7 l) connected to a vacuum pump and a pressure gauge. The petiole of the leaf was attached to a Tygon tube filled with 20 mM KCl solution filtered with a 0.22 µm membrane filter and degassed overnight via a vacuum pump. The other end of the Tygon tube was placed into a KCl solution container, which was placed on a balance (model AG204 ± 0.1 mg sensitivity; Mettler Toledo Japan, Tokyo, Japan). Five vacuum levels in the range 0.035–0.065 MPa were applied (0.005 MPa interval). At each pressure level, the mass of water on the balance was logged every 30 or 60 s at stable flow rates (*F*). *K*_leaf_ was calculated as the slope of the flow rate against vacuum pressure, normalized for leaf area, which was determined by a digital image analysis after scanning. Leaves were illuminated with ≈500 µmol m^−2^ s^−1^ photosynthetically active radiation (PAR), provided as white and blue (9 : 1) LED light, starting one hour before measurements and lasting until the measurements were complete. The room temperature was maintained at ≈25°C.

### (b) Major vein blockage experiment

Vein blockage experiments were conducted according to Nardini & Salleo [25] in three species with different life forms—the evergreen tree *Q. glauca*, the deciduous tree *Q. serrata* and the deciduous vine *P. lobata*—between August and September 2003. In the evening of the day before the *K*_leaf_ measurements, sunlit shoots were sampled, immediately recut under water and then transported to the laboratory. The cut ends of the shoots were kept in the water until the measurements started. Three different patterns of vein cuttings were performed with a fresh razor blade (figure 1): (1) the midrib was cut at one-quarter of the length (cut A); (2) the midrib was cut at three points, and all second-order veins were cut at approximately 4 mm from their base (cut B); and (3) the midrib was cut at approximately 2 mm from the base in *Q. glauca* and *Q. serrata*, and the midrib and the two large second-order veins nearest the lamina base were cut at approximately 2 mm from the base in *P. lobata* (cut C). In cut A, water flow was interrupted at a quarter of the midrib, but water could pass through the lateral secondary veins connected to up to a quarter of the midrib. In cut B, water flow through all second-order veins was interrupted, but water could flow through a quarter of the midrib and via minor veins and outside-xylem pathways. In cut C, water inlet into the leaf was extremely limited, and the only path for water flow was through minor veins and outside-xylem pathways. After vein cutting, all cut surfaces were immediately sealed with cyanoacrylate to prevent water flow [25]. On the next day, *K*_leaf_ of the treated and untreated leaves was measured. Five or six leaves per treatment per species were measured, except for four leaves for cut C in *Q. serrata*.

To investigate the effects of vein blockage on stomatal conductance (*g*_s_) *in situ*, treatments were conducted on intact leaves of the trees and vine in the field during the evening. The next day, treated and untreated leaves were measured (10.00–14.00) for *g*_s_ and leaf water potential (*Ψ*_leaf_) using a steady-state porometer (Li1600, Li-Cor, Lincoln, USA) and a pressure chamber (Soilmoisture Equipment, Santa Barbara, USA). *g*_s_ was measured at the distal third of the lamina. Six to eight leaves per treatment per species were measured. The percentage loss of conductance (PLC) of *K*_leaf_ and *g*_s_ was calculated as follows:

2.1 $$\mathrm{PLC}=100\times \left(1\text{–}\frac{K_{\mathrm{leaf}}\;\mathrm{or}\;g_{s}\;\mathrm{of}\;\mathrm{treated}\;\mathrm{leaves}}{K_{\mathrm{leaf}}\;\mathrm{or}\;g_{s}\;\mathrm{of}\;\mathrm{control}\;(i.e.\;\mathrm{untreated}\;\mathrm{leaves})\;}\right).$$

Leaf water relations were analysed by the pressure–volume technique [34], and the leaf water potential at the turgor loss point (*Ψ*_w.tlp_), osmotic potential at full turgor (*Ψ*_s.sat_) and bulk modulus of elasticity (*ε*_max_) were calculated [35]. Sunlit shoots were collected in the evening and rehydrated overnight. Seven to ten leaves of each species were measured.

### (c) Aquaporin inhibition experiment

The inhibition of water flow through aquaporins was performed using the common aquaporin inhibitor HgCl_2_ in all study species between August and September 2009. Sunlit shoots were sampled before 09.00, immediately recut under water, and transported to the laboratory. A leaf was connected to a Tigon tube filled with degassed and filtered 20 mM KCl solution, and *g*_s_ was monitored using a portable open gas exchange system (Li-6400, Li-Cor, Lincoln, USA) equipped with a 2 × 3 cm broadleaf chamber and an integrated light source (Li-6400-02B; Li-Cor) until *g*_s_ stabilized. The chamber conditions were set as follows: 700 µmol m^−2^ s^−1^ PAR, 370 µmol mol^−1^ CO_2_, 25°C block temperature and roughly 60% relative humidity. After *g*_s_ stabilization, the leaf was removed and connected to a Tigon tube filled with a degassed and filtered 20 mM KCl + 0.2 mM HgCl_2_ solution (this HgCl_2_ concentration can fully inhibit the water channel function of aquaporin [23,26]), and *g*_s_ was re-monitored and measured under the same chamber conditions. The HgCl_2_ solution was perfused to the leaf by a transpiration stream for 1 h, and then, *g*_s_ was recorded. No infiltration of water into intercellular spaces was observed during the *g*_s_ measurement. After the *g*_s_ measurement with HgCl_2_, the leaf was placed in the vacuum chamber, and *K*_leaf_ was measured. We repeated the same procedure with a 20 mM KCl solution without 0.2 mM HgCl_2_ and examined whether the experimental water inflow to the leaves over the course of 1 h affected *g*_s_ (*n* = 5 per species); no significant difference was observed between the *g*_s_ readings before and after perfusion (*p* = 0.37 − 0.95, paired *t*-test). After the *g*_s_ measurement, *K*_leaf_ was measured for the control. The PLC of *K*_leaf_ and *g*_s_ was calculated by equation (2.1); *g*_s_ before the HgCl_2_ treatment served as the control. After the experiment, whole leaf area and dry mass were measured, and leaf dry mass per area (LMA) was calculated.

### (d) Leaf vein density and vessel area

The major and minor vein densities and largest vessel area in the midrib were measured using ImageJ 1.43u (National Institutes of Health, USA https://imagej.nih.gov/ij/). The major vein density (midrib and second- and third-order veins) was measured from a whole leaf image taken by a digital scanner. Minor vein density was measured from a digital image of a 4–10 mm^2^ leaf section at the centre of the lamina taken by a digital camera attached to a light microscope. We did not apply a leaf clearing treatment for the minor vein observation because all study species had heterobaric leaves with bundle sheath extensions, which enabled us to observe the highest order veins (5th) from fresh leaves [36]. The largest vessel area at the base of the midrib was measured from a digital image of the section of the midrib, which was fixed in a formalin-acetic acid-alcohol solution. Five to seven leaves per species were measured.

### (e) Statistical analysis

All statistical analyses were performed with R v. 3.2.2 [37] at a level of statistical significance of *α* = 0.05. One-way analysis of variance (ANOVA) with a post hoc Tukey test was used to test the differences in the major and minor vein density, largest vessel area in the midrib, leaf area, LMA and leaf water relation parameters among species and in *K*_leaf_, *g*_s_ and *Ψ*_leaf_ among the vein blockage treatment within a species. Two-way ANOVA was used to test differences in *K*_leaf_, *g*_s_ and *Ψ*_leaf_ among the species and veins or aquaporin inhibition treatments. Weibull functions were fitted to the relationships between *Ψ*_leaf_ and *g*_s_ with the R package fitplc [38]. The maximum *g*_s_ in each species was calculated from the control leaves to fit a Weibull function.

## 3. Results

### (a) Leaf properties in the study species

Among the studied species, *P. lobata*, a deciduous vine with a pinnipalmate venation pattern, had the lowest LMA and density of major and minor veins, the largest leaf area and vessel area in the midrib (ANOVA with post hoc Tukey test; electronic supplementary material, table S1). *Quercus glauca*, an evergreen tree, had the highest densities of the major and minor veins. LMA was higher in the evergreen trees than in the deciduous trees, while there was no distinct difference between evergreen and deciduous trees for the other leaf properties (electronic supplementary material, table S1).

Among the three species tested in the vein blockage experiment, *Ψ*_w.tlp_ and *Ψ*_s.sat_ were higher in *P. lobata* than in *Q. glauca* and *Q. serrata* (electronic supplementary material, table S2). The value of *ε*_max_ was highest in the evergreen *Q. glauca* and lowest in the vine *P. lobata*.

### (b) Leaf major vein blockage experiment

**Figure 2.**
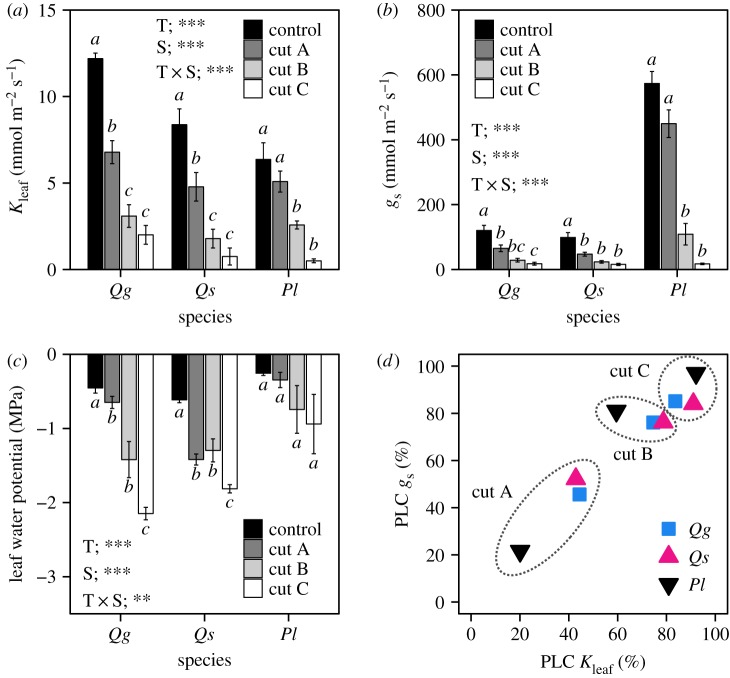
Effects of vein blockage on (*a*) leaf hydraulic conductance (*K*_leaf_, measured in the laboratory), (*b*) stomatal conductance (*g*_s,_ measured in the field), (*c*) leaf water potential at midday (measured in the field) and (*d*) the relationship between PLC of *K*_leaf_ and *g*_s_. *Qg*: *Quercus glauca* (evergreen tree); *Qs*: *Q. serrata* (deciduous tree); *Pl*: *Pueraria lobata* (deciduous vine). Bars represent ± s.e.m. (*K*_leaf_; *n* = 4–6, *g*_s_ and leaf water potential; *n* = 6–8). Asterisks in panels (*a*–*c*) represent the results of the two-way ANOVA (T, blockage treatment; S, species; T × S, interaction; ***p < 0.001, **p < 0.01). Letters in panels (*a*–*c*) represent significant differences among blockage treatments within species (Tukey–Kramer multiple comparison, *p* < 0.05).

The three types of vein blockage caused similar decreases in *K*_leaf_ and *g*_s_ in the three species (figure 2*a*,*b*), although statistical significance was not observed between some blockage treatments. Of the cut types, cut A had the least effect on *K*_leaf_ and *g*_s_, and the PLCs of the *K*_leaf_ and *g*_s_ were 40–50% in *Q. glauca* and *Q. serrata* and ≈20% in *P. lobata* (figure 2*d*). Cut B had an intermediate effect, and the PLCs of *K*_leaf_ and *g*_s_ were 80% for all three species (except for *K*_leaf_ in *P. lobata*, which was approx. ≈60%). Cut C had the greatest effect, and the PLCs of *K*_leaf_ and *g*_s_ were ≈90% in all species. Leaf water potential at midday (*Ψ*_leaf_) also tended to decline in the order of control < cut A < cut B < cut C in *Q. glauca* (figure 2*c*); however, the effect of vein blockage was unclear in comparison to the effects on *K*_leaf_ and *g*_s_ (i.e. the orders of cut A and cut B were reversed in *Q. serrata*, and there was no significant difference in *Ψ*_leaf_ between the cutting treatments in *P. lobata*).

**Figure 3.**
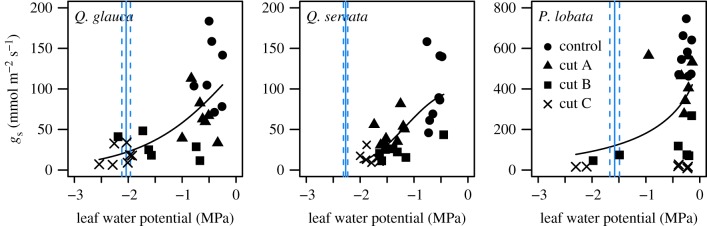
Relationships between leaf water potential and stomatal conductance (*g*_s_) at midday in vein blockage experiments. Blue solid and dashed lines represent the mean ± s.e.m. of leaf water potential, respectively, at the turgor loss point in each species (*n* = 7–10). Weibull functions are fitted.

In *Q. glauca*, *g*_s_ gradually decreased with decreasing *Ψ*_leaf_, which decreased below turgor loss points in leaves with the cut C treatment (figure 3). In *Q. serrata*, *g*_s_ also gradually decreased with decreasing *Ψ*_leaf_, but *Ψ*_leaf_ did not decrease below the turgor loss point in any leaves. In *P. lobata*, *g*_s_ rapidly decreased in most leaves even with cut C, where *Ψ*_leaf_ was more than –0.5 MPa, and almost all leaves maintained *Ψ*_leaf_ above the turgor loss point.

### (c) Aquaporin inhibition experiment

**Figure 4.**
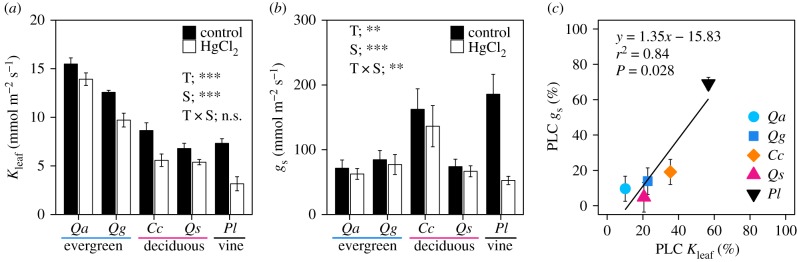
Effects of aquaporin inhibition by HgCl_2_ on (*a*) leaf hydraulic conductance (*K*_leaf_) and (*b*) stomatal conductance (*g*_s_) and (*c*) the relationship between PLC of *K*_leaf_ and *g*_s_ by aquaporin blockage. Evergreen trees: *Qa*, *Quercus acuta*; *Qg*, *Q. glauca*. Deciduous trees: Cc, *Castanea crenata; Qs*, *Q. serrata*. Deciduous vine: *Pl*, *Pueraria lobata*. Bars represent ± s.e.m. (*K*_leaf_: *n* = 5–7; *g*_s_: *n* = 5–7). Asterisks in panels (*a*) and (*b*) represent results of two-way ANOVA (T, blockage treatment; S, species; T × S, interaction; ***p < 0.001, **p < 0.01, n.s., *p* > 0.05).

*K*_leaf_ and *g*_s_ were significantly affected by aquaporin inhibition in all species (figure 4*a*,*b*). The effects of aquaporin blockage on *K*_leaf_ and *g*_s_ were relatively small in the evergreen and deciduous trees, with a 10–35% reduction in *K*_leaf_ and 5–19% in *g*_s_ on average, but the effects were higher in the deciduous vine *P. lobata*, with a 57% reduction in *K*_leaf_ and 69% in *g*_s_. There was a significant interaction between species and HgCl_2_ treatment for *g*_s_ (figure 4*b*). Pooling the data by species, the PLC of *K*_leaf_ by aquaporin blockage was positively correlated with the PLC of *g*_s_ (*r*^2^ = 0.84, *p* = 0.028; figure 4*c*). In evergreen and deciduous tree species, the PLC of *K*_leaf_ tended to be higher than the PLC of *g*_s_, whereas in the deciduous vine *P. lobata*, the PLC of *K*_leaf_ was lower than the PLC of *g*_s_.

**Figure 5.**
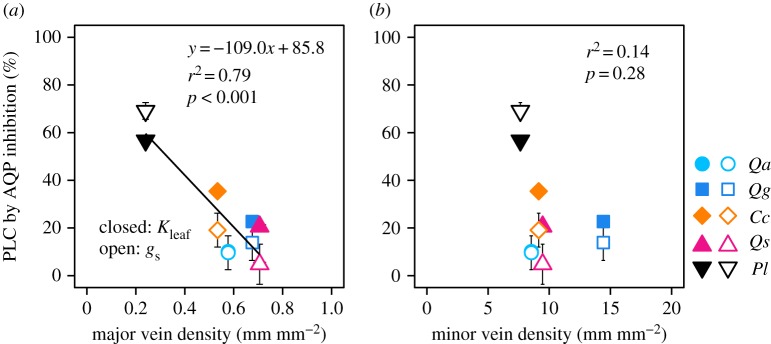
Relationships between PLC by aquaporin inhibition and (*a*) major vein density and (*b*) minor vein density. Species abbreviations are the same as in figure 4. Closed symbols: *K*_leaf_, open symbols: *g*_s_. Bars represent ± s.e.m. (*n* = 5–7). (Online version in colour.)

The PLCs of *K*_leaf_ and *g*_s_ by aquaporin blockage were significantly negatively correlated with major vein density, but not correlated with minor vein density (i.e. species with lower major vein density experienced greater effects on *K*_leaf_ and *g*_s_; figure 5).

## 4. Discussion

The results of the major vein blockage treatment showed a similar tendency to those found in previous studies [16,25]. When water flow was blocked a quarter of the way along the midrib in the cut A treatment, *P. lobata* with large and long second-order veins extending from the base of the midrib to the outer margins of the leaf (i.e. pinnipalmate venation, figure 1*b*) showed a lower reduction in *K*_leaf_ and *g*_s_ than that in *Q. glauca* and *Q. serrata* with pinnate leaves (which have a single midrib). A minimal impact against a blockage of the middle position of the midrib has also been reported in palmate leaves, which have three or more primary veins at the base of the leaf blade [16]. In addition, recent studies have shown that in comparison with minor veins, lower-order veins with larger conduits were more vulnerable to embolism during leaf dehydration, suggesting a trade-off between efficiency and safety in the leaf venation network, where larger conduits can transport water more efficiently but are more vulnerable to losing their hydraulic function by embolism [15,39,40]. Therefore, leaves with the largest vessel in *P. lobata* would be most vulnerable to xylem embolism; that is, they would be likely to experience hydraulic failure, such as in the cut B and cut C treatments during leaf dehydration. In leaves with the cut C treatment, a higher PLC of *K*_leaf_ and *g*_s_ was observed in *P. lobata* with a lower density of minor veins, and a lower PLC was observed in *Q. glauca* with a higher density of minor veins (figure 2*d*). This result may have partially occurred due to the longer outside-xylem pathway with high hydraulic resistance from the lower minor vein density in *P. lobata*. Our results suggest that the specific difference in venation properties, such as a large venation arrangement, minor vein density and vessel area, can affect a *g*_s_ decline due to midrib damage [16] and vein embolism during dehydration.

While aquaporin inhibition by the HgCl_2_ solution reduced both *K*_leaf_ and *g*_s_ in all species studied (figure 4*a*,*b*), the extent of the decrease varied among species, ranging from a 10% to 57% reduction in *K*_leaf_ and a 5% to 69% reduction in *g*_s_. Similar reductions in *K*_leaf_ by a HgCl_2_ treatment have been reported in other species: *Populus* (22–67% reduction [41,42]), deciduous trees (32–60% reduction [43]), *Vitis* (25 and 61% reduction [21]), and *Arabidopsis* (50% reduction [44]). A physiological mechanism of stomatal closure mediated by aquaporin during drought was proposed; drought-induced abscisic acid (ABA) in the xylem sap flows into the laminae from the petiole, and then, ABA downregulates the activity of aquaporins in bundle sheath cells surrounding veins, leading to reduced *K*_leaf_. In turn, this results in rapid *Ψ*_leaf_ reduction and consequently in stomatal closure [44–46]. Thus, ABA can have dual effects on stomatal closure: a biochemical direct effect on the guard cells and an indirect hydraulic effect through aquaporin-mediated decline in *K*_leaf_ [45]. Since aquaporin inhibition experiments would cause effects analogous to aquaporin downregulation, our results, in which species with higher reductions in *K*_leaf_ by the aquaporin blocker HgCl_2_ tended to have a greater reduction in *g*_s_ (figure 4*c*), would support the aquaporin-mediated and hydraulically induced stomatal closure.

Notably, the specific difference in aquaporin activity within a leaf might involve differences in water-use strategies via isohydric or anisohydric stomatal regulation [45,47]. Isohydric plants rapidly close their stomata under drought, which results in the maintenance of a constant *Ψ*_leaf_ and avoids the risk of catastrophic cavitation, as observed in *P. lobata* (figure 3). By contrast, anisohydric plants slowly close their stomata during drought, resulting in a lower *Ψ*_leaf_, which, although increasing the risk of cavitation, maintains photosynthetic production [48], as observed in *Q. glauca* and *Q. serrata*. Scoffoni *et al.* reported that water flow through the outside-xylem pathway was more vulnerable to drought than that through leaf vein xylem in eight species with diverse phylogenies, origins, drought tolerances and life forms, and that reduced aquaporin activity would be the main determinant of the decline in outside-xylem conductance from a model analysis [49]. Therefore, our results that large declines in *K*_leaf_ and *g*_s_ following aquaporin inhibition were found in isohydric *P. lobata* and that small declines in *K*_leaf_ and *g*_s_ were found in anisohydric *Q. acuta* and *Q. serrata* support the hypothesis that hydraulic regulation through aquaporin downregulation might be involved in specific water-use strategies, such as isohydric and anisohydric stomatal regulation. In addition, the low *ε*_max_ in *P. lobata* (electronic supplementary material, table S2) would be susceptible to mesophyll shrinkage during drought, possibly resulting in rapid reduction in *K*_leaf_ via a reduction in hydraulic conductivity in another outside-xylem pathway, a mesophyll route [50]. The high vulnerability of the outside-xylem pathway could lead to rapid stomatal closure that would protect against catastrophic hydraulic failure in the stem xylem during drought, which would induce plant death [49]. Because the stem of *P. lobata* can be highly sensitive to drought-induced cavitation, such as when the mean cavitation pressure is –0.58 MPa [51], the rapid stomatal closure involved in aquaporin as well as mesophyll shrinkage would protect the plant from not only major vein embolism but also stem embolism and subsequent plant death during drought.

In comparison with minor veins, major veins potentially have greater water transport capacity and a greater carbon cost, and the higher major vein density potentially contributes to higher *K*_leaf_ and gas exchange rates under moist conditions [52]. We found that species with lower major vein density had a higher PLC for *K*_leaf_ and *g*_s_ due to aquaporin inhibition in a rehydrated leaf (figure 5*a*). This result may suggest that a trade-off function exists between vein architecture (apoplastic pathway) and aquaporin activity (cell-to-cell pathway) with respect to leaf hydraulics. That is, species with higher costs of the major veins can supply enough water to the whole lamina, such that they do not necessarily need to significantly increase the outside-xylem hydraulic conductivity by the aquaporins, whereas species with lower costs of the major veins need to increase the outside-xylem hydraulic conductivity by increasing the aquaporin expression level in a hydrated leaf. Higher major vein density can influence higher LMA and longer leaf spans across species [7]. In addition, high aquaporin dependence on *K*_leaf_ in *P. lobata* may be related to the life form of the species. Enhanced aquaporin expression and activity in roots and shoots promote an increase in whole-plant hydraulic conductance, thus mitigating *Ψ*_leaf_ decline and facilitating stomatal opening [47]. A great extent of aquaporin regulation in *P. lobata* would be advantageous for plastic and rapid responses of plant water use to the changing environment because *P. lobata* is a vine species; thus, in comparison with woody species, a vine can easily move to a new expanding environment by extending the new shoots. More studies are needed to clarify the underlying constraint in the trade-off relationship related to leaf hydraulics.

Overall, our results suggest that specific differences in leaf venation architecture, especially major vein density and arrangement, and in the activity of leaf aquaporin, can affect *K*_leaf_ and *g*_s_, potentially due to their ecological properties, such as growth form, isohydry/anisohydry and vulnerability to embolism, possibly providing a trade-off function between vein architecture and aquaporin activity. Leaf vulnerability to embolism is one of the most central traits for plant drought tolerance [53]; therefore, regulation of *K*_leaf_ by aquaporins and tolerance of embolism in major veins (and leaf venation architecture) are important traits that determine drought tolerance in angiosperms. Further studies of aquaporins in relation to leaf hydraulics across a wider range of phylogeny and species will provide a deeper understanding of specific drought tolerance and drought-induced vegetation mortality.

## Supplementary Material

Supplementary tables

## Supplementary Material

Supplementary data sheet

## Data accessibility

All data generated or analysed during this study are included in this published article and its electronic supplementary material.

## References

[1] Tyree MT, Ewers FW 1991 The hydraulic architecture of trees and other woody plants. New Phytol. 119, 345–360. (10.1111/j.1469-8137.1991.tb00035.x)

[2] Sperry JS 2000 Hydraulic constraints on plant gas exchange. Agric. For. Meteorol. 104, 13–23. (10.1016/s0168-1923(00)00144-1)

[3] McCulloh KA, Johnson DM, Petitmermet J, McNellis B, Meinzer FC, Lachenbruch B 2015 A comparison of hydraulic architecture in three similarly sized woody species differing in their maximum potential height. Tree Physiol. 35, 723–731. (10.1093/treephys/tpv035) 25972291

[4] Sack L, Cowan PD, Jaikumar N, Holbrook NM 2003 The ‘hydrology’ of leaves: co-ordination of structure and function in temperate woody species. Plant Cell Environ. 26, 1343–1356. (10.1046/j.0016-8025.2003.01058.x)

[5] Sack L, Holbrook NM 2006 Leaf hydraulics. Annu. Rev. Plant Biol. 57, 361–381. (10.1146/annurev.arplant.56.032604.144141) 16669766

[6] Brodribb TJ, Feild TS, Jordan GJ 2007 Leaf maximum photosynthetic rate and venation are linked by hydraulics. Plant Physiol. 144, 1890–1898. (10.1104/pp.107.101352) 17556506 PMC1949879

[7] Sack L, Scoffoni C, John GP, Poorter H, Mason CM, Mendez-Alonzo R, Donovan LA 2013 How do leaf veins influence the worldwide leaf economic spectrum? Review and synthesis. J. Exp. Bot. 64, 4053–4080. (10.1093/jxb/ert316) 24123455

[8] Nardini A, Luglio J, Thompson K 2014 Leaf hydraulic capacity and drought vulnerability: possible trade-offs and correlations with climate across three major biomes. Funct. Ecol. 28, 810–818. (10.1111/1365-2435.12246)

[9] Scoffoni C, Chatelet DS, Pasquet-Kok J, Rawls M, Donoghue MJ, Edwards EJ, Sack L 2016 Hydraulic basis for the evolution of photosynthetic productivity. Nat. Plants 2, 16072 (10.1038/nplants.2016.72) 27255836

[10] Taneda H, Kandel DR, Ishida A, Ikeda H 2016 Altitudinal changes in leaf hydraulic conductance across five *Rhododendron* species in eastern Nepal. Tree Physiol. 36, 1272–1282. (10.1093/treephys/tpw058) 27417514

[11] Roth-Nebelsick A, Uhl D, Mosbrugger V, Kerp H 2001 Evolution and function of leaf venation architecture: a review. Ann. Bot. 87, 553–566. (10.1006/anbo.2001.1391)

[12] Sack L, Frole K 2006 Leaf structural diversity is related to hydraulic capacity in tropical rain forest trees. Ecology 87, 483–491. (10.1890/05-0710) 16637372

[13] Sack L, Tyree MT, Holbrook NM 2005 Leaf hydraulic architecture correlates with regeneration irradiance in tropical rainforest trees. New Phytol. 167, 403–413. (10.1111/j.1469-8137.2005.01432.x) 15998394

[14] Johnson DM, Meinzer FC, Woodruff DR, McCulloh KA 2009 Leaf xylem embolism, detected acoustically and by cryo-SEM, corresponds to decreases in leaf hydraulic conductance in four evergreen species. Plant Cell Environ. 32, 828–836. (10.1111/j.1365-3040.2009.01961.x) 19220781

[15] Brodribb TJ, Skelton RP, McAdam SA, Bienaime D, Lucani CJ, Marmottant P 2016 Visual quantification of embolism reveals leaf vulnerability to hydraulic failure. New Phytol. 209, 1403–1409. (10.1111/nph.13846) 26742653

[16] Sack L, Dietrich EM, Streeter CM, Sanchez-Gomez D, Holbrook NM 2008 Leaf palmate venation and vascular redundancy confer tolerance of hydraulic disruption. Proc. Natl Acad. Sci. USA 105, 1567–1572. (10.1073/pnas.0709333105) 18227511 PMC2234185

[17] Prado K, Maurel C 2013 Regulation of leaf hydraulics: from molecular to whole plant levels. Front. Plant Sci. 4, 255 (10.3389/fpls.2013.00255) 23874349 PMC3711007

[18] Li G, Santoni V, Maurel C 2014 Plant aquaporins: roles in plant physiology. Biochim. Biophys. Acta 1840, 1574–1582. (10.1016/j.bbagen.2013.11.004) 24246957

[19] Cochard H, Venisse JS, Barigah TS, Brunel N, Herbette S, Guilliot A, Tyree MT, Sakr S 2007 Putative role of aquaporins in variable hydraulic conductance of leaves in response to light. Plant Physiol. 143, 122–133. (10.1104/pp.106.090092) 17114274 PMC1761984

[20] Liu J, Equiza MA, Navarro-Rodenas A, Lee SH, Zwiazek JJ 2014 Hydraulic adjustments in aspen (*Populus tremuloides*) seedlings following defoliation involve root and leaf aquaporins. Planta 240, 553–564. (10.1007/s00425-014-2106-2) 24957702

[21] Pou A, Medrano H, Flexas J, Tyerman SD 2013 A putative role for TIP and PIP aquaporins in dynamics of leaf hydraulic and stomatal conductances in grapevine under water stress and re-watering. Plant Cell Environ. 36, 828–843. (10.1111/pce.12019) 23046275

[22] Perez-Martin A, Michelazzo C, Torres-Ruiz JM, Flexas J, Fernandez JE, Sebastiani L, Diaz-Espejo A 2014 Regulation of photosynthesis and stomatal and mesophyll conductance under water stress and recovery in olive trees: correlation with gene expression of carbonic anhydrase and aquaporins. J. Exp. Bot. 65, 3143–3156. (10.1093/jxb/eru160) 24799563 PMC4071832

[23] Laur J, Hacke UG 2014 The role of water channel proteins in facilitating recovery of leaf hydraulic conductance from water stress in *Populus trichocarpa*. PLoS ONE 9, e111751 (10.1371/journal.pone.0111751) 25406088 PMC4236056

[24] Scoffoni C, Sack L, Ort D 2017 The causes and consequences of leaf hydraulic decline with dehydration. J. Exp. Bot. 68, 4479–4496. (10.1093/jxb/erx252) 28981777

[25] Nardini A, Salleo S 2003 Effects of the experimental blockage of the major veins on hydraulics and gas exchange of *Prunus laurocerasus* L. leaves. J. Exp. Bot. 54, 1213–1219. (10.1093/jxb/erg130) 12654872

[26] Nardini A, Salleo S, Andri S 2005 Circadian regulation of leaf hydraulic conductance in sunflower (*Helianthus annuus* L. cv Margot). Plant Cell Environ. 28, 750–759. (10.1111/j.1365-3040.2005.01320.x)

[27] Tyree MT, Nardini A, Salleo S, Sack L, El Omari B 2005 The dependence of leaf hydraulic conductance on irradiance during HPFM measurements: any role for stomatal response? J. Exp. Bot. 56, 737–744. (10.1093/jxb/eri045) 15582928

[28] Voicu MC, Zwiazek JJ, Tyree MT 2008 Light response of hydraulic conductance in bur oak (*Quercus macrocarpa*) leaves. Tree Physiol. 28, 1007–1015. (10.1093/treephys/28.7.1007) 18450565

[29] Baaziz KB, Lopez D, Rabot A, Combes D, Gousset A, Bouzid S, Cochard H, Sakr S, Venisse JS 2012 Light-mediated Kleaf induction and contribution of both the PIP1s and PIP2s aquaporins in five tree species: walnut (*Juglans regia*) case study. Tree Physiol. 32, 423–434. (10.1093/treephys/tps022) 22544048

[30] Harayama H, Ishida A, Yoshimura J 2016 Overwintering evergreen oaks reverse typical relationships between leaf traits in a species spectrum. R. Soc. open sci. 3, 160276 (10.1098/rsos.160276) 27493781 PMC4968473

[31] Kolb KJ, Sperry JS, Lamont BB 1996 A method for measuring xylem hydraulic conductance and embolism in entire root and shoot systems. J. Exp. Bot. 47, 1805–1810. (10.1093/jxb/47.11.1805)

[32] Nardini A, Tyree MT, Salleo S 2001 Xylem cavitation in the leaf of *Prunus laurocerasus* and its impact on leaf hydraulics. Plant Physiol. 125, 1700–1709. (10.1104/pp.125.4.1700) 11299351 PMC88827

[33] Lo Gullo MA, Nardini A, Trifilo P, Salleo S 2003 Changes in leaf hydraulics and stomatal conductance following drought stress and irrigation in *Ceratonia siliqua* (carob tree). Physiol. Plant. 117, 186–194. (10.1034/j.1399-3054.2003.00038.x)

[34] Tyree MT, Hammel HT 1972 The measurement of the turgor pressure and the water relations of plants by the pressure-bomb technique. J. Exp. Bot. 23, 267–282. (10.1093/jxb/23.1.267)

[35] Schulte PJ, Hinckley TM 1985 A comparison of pressure–volume curve data analysis techniques. J. Exp. Bot. 36, 1590–1602. (10.1093/jxb/36.10.1590)

[36] Kawai K, Okada N, Watling J 2016 How are leaf mechanical properties and water-use traits coordinated by vein traits? A case study in Fagaceae. Funct. Ecol. 30, 527–536. (10.1111/1365-2435.12526)

[37] R Core Team. 2015 R: A language and environment for statistical computing. 3.2.2. edn Vienna, Austria: R Foundation for Statistical Computing.

[38] Duursma R, Choat B 2017 fitplc: an R package to fit hydraulic vulnerability curves. J. Plant Hydraulics 4, 002 (10.20870/jph.2017.e002)

[39] Brodribb TJ, Bienaime D, Marmottant P 2016 Revealing catastrophic failure of leaf networks under stress. Proc. Natl Acad. Sci. USA 113, 4865–4869. (10.1073/pnas.1522569113) 27071104 PMC4855591

[40] Scoffoni C, Albuquerque C, Brodersen CR, Townes SV, John GP, Cochard H, Buckley TN, McElrone AJ, Sack L 2017 Leaf vein xylem conduit diameter influences susceptibility to embolism and hydraulic decline. New Phytol. 213, 1076–1092. (10.1111/nph.14256) 27861926

[41] Lopez D, Venisse JS, Fumanal B, Chaumont F, Guillot E, Daniels MJ, Cochard H, Julien JL, Gousset-Dupont A 2013 Aquaporins and leaf hydraulics: poplar sheds new light. Plant Cell Physiol. 54, 1963–1975. (10.1093/pcp/pct135) 24058149

[42] Voicu MC, Zwiazek JJ 2010 Inhibitor studies of leaf lamina hydraulic conductance in trembling aspen (*Populus tremuloides* Michx.) leaves. Tree Physiol. 30, 193–204. (10.1093/treephys/tpp112) 20022867

[43] Aasamaa K, Sõber A 2005 Seasonal courses of maximum hydraulic conductance in shoots of six temperate deciduous tree species. Funct. Plant Biol. 32, 1077–1087. (10.1071/fp05088) 32689203

[44] Shatil-Cohen A, Attia Z, Moshelion M 2011 Bundle-sheath cell regulation of xylem-mesophyll water transport via aquaporins under drought stress: a target of xylem-borne ABA? Plant J. 67, 72–80. (10.1111/j.1365-313X.2011.04576.x) 21401747

[45] Pantin F, Monnet F, Jannaud D, Costa JM, Renaud J, Muller B, Simonneau T, Genty B 2013 The dual effect of abscisic acid on stomata. New Phytol. 197, 65–72. (10.1111/nph.12013) 23106390

[46] Sade N et al. 2014 The role of plasma membrane aquaporins in regulating the bundle sheath-mesophyll continuum and leaf hydraulics. Plant Physiol. 166, 1609–1620. (10.1104/pp.114.248633) 25266632 PMC4226360

[47] Maurel C, Verdoucq L, Rodrigues O 2016 Aquaporins and plant transpiration. Plant Cell Environ. 39, 2580–2587. (10.1111/pce.12814) 27497047

[48] Tardieu F, Simonneau T 1998 Variability among species of stomatal control under fluctuating soil water status and evaporative demand: modelling isohydric and anisohydric behaviours. J. Exp. Bot. 49, 419–432. (10.1093/jxb/49.Special_Issue.419)

[49] Scoffoni C, Albuquerque C, Brodersen CR, Townes SV, John GP, Bartlett MK, Buckley TN, McElrone AJ, Sack L 2017 Outside-xylem vulnerability, not xylem embolism, controls leaf hydraulic decline during dehydration. Plant Physiol. 173, 1197–1210. (10.1104/pp.16.01643) 28049739 PMC5291720

[50] Trifiló P, Raimondo F, Savi T, Lo Gullo MA, Nardini A 2016 The contribution of vascular and extra-vascular water pathways to drought-induced decline of leaf hydraulic conductance. J. Exp. Bot. 67, 5029–5039. (10.1093/jxb/erw268) 27388214

[51] Hacke UG, Sperry JS, Wheeler JK, Castro L 2006 Scaling of angiosperm xylem structure with safety and efficiency. Tree Physiol. 26, 689–701. (10.1093/treephys/26.6.689) 16510385

[52] Sack L, Scoffoni C 2013 Leaf venation: structure, function, development, evolution, ecology and applications in the past, present and future. New Phytol. 198, 983–1000. (10.1111/nph.12253) 23600478

[53] Bartlett MK, Klein T, Jansen S, Choat B, Sack L 2016 The correlations and sequence of plant stomatal, hydraulic, and wilting responses to drought. Proc. Natl Acad. Sci. USA 113, 13 098–13 103. (10.1073/pnas.1604088113) PMC5135344 27807136

