# Supplementary material for: Effects of major vein blockage and aquaporin inhibition on leaf hydraulics and stomatal conductance
Source: Proc Biol Sci. 2019 Jun 5;286(1904):20190799. doi: 10.1098/rspb.2019.0799 (PMC6571453; doi:10.1098/rspb.2019.0799)
Supplement: Supplementary tables [file rspb20190799supp1.docx]

**Electronic Supplementary Information (ESM)**

Effects of major vein blockage and aquaporin inhibition on leaf hydraulics and stomatal conductance

Hisanori Harayama, Mitsutoshi Kitao, Evgenios Agathokleous and Atsushi Ishida

| Supplementary table S1. Leaf architectural and anatomical traits in the study species. The data of major and minor vein density, leaf area, leaf mass per area and largest vessel area in the midrib are presented as the means ± SEM (vein density; *n* = 5–7, leaf area; *n* = 11–13, leaf mass per area; *n* = 6, vessel area; n = 6–8). Letters represent significant differences among species in each parameter (one-way ANOVA with post-hoc Tukey test, *P* < 0.05) | | | | | | | |
| --- | --- | --- | --- | --- | --- | --- | --- |
|  | **Evergreen tree** | |  | **Deciduous tree** | |  | **Deciduous vine** |
|  | ***Quercus acuta*** | ***Q. glauca*** |  | ***Castanea crenata*** | ***Q. serrata*** |  | ***Pueraria lobata*** |
| **Vein architecture type** | pinnate | pinnate |  | pinnate | pinnate |  | pinnipalmate |
| **Major vein density (mm mm^−2^)** | 0.58 ± 0.02 b | 0.68 ± 0.02 a |  | 0.53 ± 0.02 b | 0.71 ± 0.02 a |  | 0.24 ± 0.01 c |
| **Minor vein density (mm mm^−2^)** | 8.53 ± 0.23 c | 14.31 ± 0.72 a |  | 9.44 ± 0.47 bc | 9.36 ± 0.15 b |  | 7.65 ± 0.17 d |
| **Total vein density (mm mm^−2^)** | 9.10 | 14.98 |  | 9.98 | 10.06 |  | 7.89 |
| **Leaf area (cm^2^)** | 32.8 ± 1.0 bc | 45.3 ± 4.4 b |  | 52.0 ± 3.5 b | 26.6 ± 1.2 c |  | 126 ± 8.9 a |
| **Leaf mass per area (g m^−2^)** | 113.4 ± 2.2 a | 114.2 ± 2.2 a |  | 64.9 ± 1.4 c | 87.3 ± 1.9 b |  | 28.6 ± 2.7 d |
| **Largest vessel area in the midrib (µm^2^)** | 938 ± 58 b | 1192 ± 40 b |  | 970 ± 66 b | 1100 ± 103 b |  | 3083 ± 158 a |

| Supplementary table S2. Leaf water potential at turgor loss point (*Ψ*_w.tlp_), leaf osmotic potential at full turgor (*Ψ*_s.sat_) and maximum bulk modulus of elasticity (*ε*_max_) calculated on the basis P-V curve. The data are presented as means ± SEM (*n* = 7–10). Letters represent significant differences among species in each parameter (one-way ANOVA with post-hoc Tukey test, *P* < 0.05). | | | |  |
| --- | --- | --- | --- | --- |
|  | ***Quercus glauca*** | ***Q. serrata*** | ***Pueraria lobata*** | |
| ***Ψ*_w.tlp_ (MPa)** | –2.04 ± 0.08 b | –2.27 ± 0.04 b | –1.58 ± 0.09 a | |
| ***Ψ*_s.sat_ (MPa)** | –1.83 ± 0.07 b | –1.92 ± 0.04 b | –1.33 ± 0.08 a | |
| ***ε*_max_ (MPa)** | 18.2 ± 1.6 a | 12.9 ± 1.1 b | 8.8 ± 0.6 c | |
